# Comparison of the Spasmolytic Effects of Jakyak-Gamcho Decoctions Derived via Different Extractants

**DOI:** 10.1155/2015/270380

**Published:** 2015-10-11

**Authors:** Dongwook Kwak, Changwoo Lee, Inseong Kong, JaeChul Lee, Donghee Choi, Changsu Na

**Affiliations:** ^1^Korean Medical College, Dongshin University, Naju 520-714, Republic of Korea; ^2^Mibyeong Research Center, Korea Institute of Oriental Medicine, Daejeon 305-811, Republic of Korea; ^3^Department of Acupoint and Meridian, Korean Medical College, Dongshin University, Naju 520-714, Republic of Korea

## Abstract

*Aim*. To investigate whether differences in the amounts of effective index components in Jakyak-Gamcho decoctions derived via extraction with either water or ethanol were associated with differential spasmolytic effectiveness. *Methods*. The amounts of effective index components (paeoniflorin, benzoic acid, glycyrrhizin, and isoliquiritin) contained in water-extracted Jakyak-Gamcho decoction and 70% ethanol-extracted Jakyak-Gamcho decoction were compared by high-performance liquid chromatography. Muscle cramp reduction rates were compared between the two decoctions by comparing the degrees of muscle contraction, measured as the tension developed during electrical stimulation, before and 1 and 2 h after injection in rats. *Results*. The relative amounts of effective index components were, on average, about 43% higher in the 70% ethanol-extracted decoction than in the water-extracted decoction. Two hours after injection, 0.25 g/kg of 70% ethanol-extracted decoction produced a significantly greater spasmolytic effect than 0.25 g/kg of water-extracted Jakyak-Gamcho decoction or distilled water (both *p* < 0.05). *Conclusion*. Differences in the amounts of effective index components resulting from the use of different extractants were associated with differences in spasmolytic effectiveness. Hence, it may be worthwhile to investigate alternative extraction methods in terms of extraction efficiency and *in vivo* effectiveness for various herbal medicines in the future.

## 1. Introduction

Oriental medicine treatment modalities including acupuncture, moxibustion, and herbal medicines are effective for the treatment of musculoskeletal conditions [1–5]. Muscle cramping is a common musculoskeletal condition, and it is accompanied by instant intense pain [6]. Muscle cramps can be classified as true cramps, tetany, contractions, and dystonic cramps [7]. Tetany refers to a large constant contraction resulting from continuous single contractions induced by a repetitive stimulus applied to muscles. Low frequency stimulations result in incomplete tetanus that exhibits a serrated contraction curve, whereas high frequency stimulations result in longer contractions and are associated with complete tetanus exhibiting a curve with a gradual plateau [8]. The factors that cause muscle cramps are known to include intense exercise, metabolic disorders, electrolyte abnormalities, and pregnancy, but additional causes remain to be clarified [9]. Muscle relaxants and spasmolytic drugs are used to treat muscle cramps, but most are associated with side effects such as hepatotoxicity and central nervous system (CNS) depression [10].

Jakyak-Gamcho decoction is a commonly prescribed spasmolytic. It comprises Radix Paeoniae and Radix Glycyrrhizae in a 1 : 1 ratio and contains the index substances paeoniflorin, benzoylpaeoniflorin, albiflorin, glycyrrhizin, isoliquiritin, and liquiritigenin, and so forth. It is reportedly effective for the alleviation of skeletal muscle cramps caused by nervous stimulation and hemodialysis [11, 12]. In addition, it has been reported that Jakyak-Gamcho decoction can relieve intestinal cramps via smooth muscle relaxation and that it is effective for the alleviation of myodynia caused by peripheral nerve damage or chemotherapy [13–15]. Particularly, among the constituents of Jakyak-Gamcho decoction, Radix Glycyrrhizae, which contains glycyrrhizin and isoliquiritin, has a significant spasmolytic effect [9]. Although the mechanism of the muscle-relaxing effect of Jakyak-Gamcho decoction remains unclear, its complex constituents interact with each other, thereby resulting in fewer CNS side effects compared to muscle relaxants that directly act on the CNS [9]. Recently, it was also reported to be effective against extrapyramidal symptoms, a side effect of antipsychotic agents [16]. Traditionally, Jakyak-Gamcho decoction is prepared with water as the extractant. However, in terms of extraction efficiency, water may not be an optimal extractant. In fact, 70% ethanol exhibits higher extraction efficiency than water [17].

Most previous studies investigating extraction efficiencies have been limited to the assessment of changes in the levels of effective index components according to different extraction methods [17]. Studies investigating whether the different levels of effective index components resulting from different methods of extraction actually lead to differential effectiveness in practice are lacking.* In vivo* studies that can elucidate optimal methods with regard to extraction efficiency in the preparation of herbal medicines are urgently needed.

This study aimed at investigating whether differences in the levels of effective index components in Jakyak-Gamcho decoctions resulting from the use of different extractants are associated with differences in its effectiveness as a spasmolytic agent in an experimental animal model. In other words, we tested the hypothesis that the higher amounts of effective index components in 70% ethanol-extracted Jakyak-Gamcho decoction than in water-extracted decoction resulted in the greater spasmolytic effects of the former* in vivo*. We also investigated whether Jakyak-Gamcho decoction is effective under physiological conditions. To this effect, we estimated the tension of complete tetanus (pathological muscle cramp) and that of incomplete tetanus (physiological contraction) and compared them. Jakyak-Gamcho decoctions were prepared via an ultrasound-reflux extraction method using either water or 70% ethanol as the extractant and injected into the duodenum of rats to compare gastrocnemius muscle tetanus reduction rates after the induction of muscle cramps.

## 2. Materials and Methods

### 2.1. Experimental Medicines and Anesthetics

Sixty grams of Radix Paeoniae (Hwalim Natural Drug Co., Ltd., Korea) and sixty grams of Radix Glycyrrhizae (Hwalim Natural Drug Co., Ltd., Korea) were used to prepare decoctions with 1.2 L of either water or 70% ethanol as the extractant, via reflux extraction for 3 hours using an ultrasonic extractor (POWER SONIC410, Hwashin, Korea, oscillation frequency: 40 KHz, temperature: 50°C). After reflux extraction, the extracts were subjected to pressure evaporation (RV10, IKA, Germany) and lyophilization via a freeze dryer (ILShin Lab Co., Ltd., Korea) and then stored in a freezer at −80°C in powder form. The powdered extracts were dissolved in distilled water (Young Lin Instrument Co., Ltd., Korea) prior to use. Isoflurane (Hana Pharm Co., Ltd., Korea) and urethane (Sigma-Aldrich, Co., St. Louis, USA) were used as anesthetics. The experimental medicines were injected into the duodenum 1 hour before the experiment. The doses of the medicines administered were as follows: water-extracted Jakyak-Gamcho decoction, 0.25 g/kg and 0.5 g/kg i.d.; 70% ethanol-extracted Jakyak-Gamcho decoction, 0.25 g/kg and 0.5 g/kg i.d.; isoflurane (99% oxygen, 5% isoflurane), inhalation anesthesia; urethane (25%), 1.5 mL i.p. The doses of water- and 70% ethanol-extracted decoctions were chosen based on the results of a preliminary study that showed that 70% ethanol-extracted Jakyak-Gamcho decoction had a spasmolytic effect at dose of 0.25 g/kg or higher, while for the water-extracted decoction, the dose had to be 0.5 g/kg or higher. In addition, a pharmacological study of Jakyak-Gamcho decoction showed that doses of 0.5 g/kg, 1 g/kg, and 2 g/kg have no effect on general behavior or CNS function [18].

### 2.2. Experimental Animals

Four male Wistar rats aged 7-8 weeks and weighing 172.5–183.1 g were used in a preliminary experiment to determine the initiating frequency. In the actual experiment, 5 male Wistar rats aged 7-8 weeks and weighing 178.6–195.8 g were used in each group. A distilled water injection (control) group, a water-extracted Jakyak-Gamcho decoction (0.25 g/kg, 0.5 g/kg) injection group, and a 70% ethanol-extracted Jakyak-Gamcho decoction (0.25 g/kg, 0.5 g/kg) injection group were included in the study. All experimental animals were adapted in a controlled environment (24°C ± 1°C, 60% ± 5% humidity, and 12-hour light/dark cycle), and water and food (Samtako Inc., Korea) were available* ad libitum*. All experiments were approved by the Institutional Review Board for Animal Studies of Dongshin University. After the completion of experiments, the animals were killed via a cardiac injection of a lethal dose of urethane.

### 2.3. High-Performance Liquid Chromatography Analysis

The Jakyak-Gamcho decoctions derived via different solvents were subjected to high-performance liquid chromatography (HPLC) analysis to investigate differences in the levels of effective index components. HPLC machine (Hewlett Packard 1100 Series, Agilent) at the Korea Basic Science Institute (Seoul) was utilized in the analysis (Column-G1316A, DAD-G1315A, QuatPump-G1311A, ALS-G1329A, and Degasser-G1322A). Paeoniflorin, benzoic acid, glycyrrhizin, and isoliquiritin were included as effective index components. Analysis conditions were as follows: column, Kinetex C18 (4.6 × 250 mm, 5 *μ*, Phenomenex); column temperature, 25°C; eluents, (A) 0.01% phosphoric acid and (B) acetonitrile; flow rate, 1.0 mL/min; run time, 60 min; wavelengths, paeoniflorin, benzoic acid, and glycyrrhizin 230 nm and isoliquiritin 360 nm; injection volume, 10 *μ*L. Gradient details are shown in Table 1.

### 2.4. Electrical Stimulation Experiment

#### 2.4.1. Surgery

After inducing inhalation anesthesia with isoflurane, 1.5 mL of 25% urethane was injected into the right abdominal cavity of rats, to maintain anesthesia. During anesthesia and surgery, vital signs were monitored via the front legs (MouseOx Plus, Starr Life Science Corp., USA). Physiological saline solution was frequently applied to avoid drying of the surgical areas.

**Figure 1:**
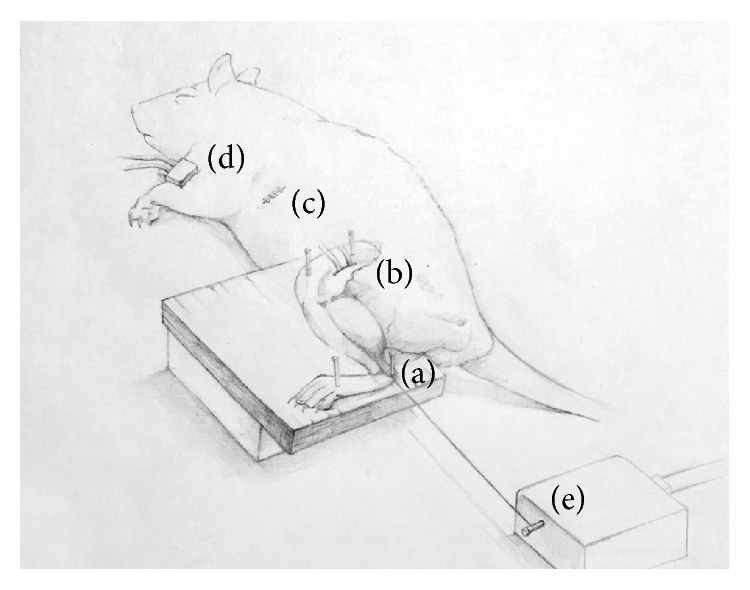
Schematic view of the experimental setup. A force transducer is attached to the gastrocnemius muscle by a silk thread on the distal Achilles tendon (a). Bipolar electrodes are attached to the tibial branch of the sciatic nerve (b). Medicine is directly injected into the duodenum (c). Vital signs are measured via the left front leg (d). Force transducer (e).

Surgery was performed in the left posterior lower limbs and abdomen. After removing hair in the surgical areas, skin from the calcaneus to the knee and from the knee to the hip joint was incised and separated from the subcutaneous muscles. The tensor fasciae latae and the gluteus maximus were then incised and removed, to expose the gastrocnemius and tibial nerves. In order to restrict electrical stimulation to the tibial nerves, potentially affected peroneal and sural nerves were incised and removed. In addition, a thin plastic film was placed underneath the exposed tibial nerve to prevent electrical stimulation from being transferred to peripheral tissues. The gastrocnemius muscle, a target for tension measurements, was located and separated and the soleus muscles attached underneath it were removed. The legs undergoing surgery were fixed to the operating table with pins, to induce isometric contraction. Pins were fixed to the left femur, knee, tibia, calcaneus, and soles (see Figure 1).

Surgery was also performed on the abdomen, to facilitate the administration of medicine. A 1.5 cm horizontal incision was made in the abdomen, and the duodenum was located for the injection of medicine. After the injection of medicine, the incision was closed using a stapler for animal surgery (Leica Biosystems Richmond Inc., USA).

#### 2.4.2. Electrical Stimulation and Muscle Cramp Induction

Only complete tetanus was deemed to constitute muscle cramping, and incomplete tetanus was deemed to constitute physiological muscle contraction. Then, the initiating frequency where complete tetanus is induced varies for each individual rat, so a preliminary study was performed to ascertain some foundation parameters. In the preliminary study, electrical stimulation of 20, 40, 60, or 70 Hz was applied for 5 seconds at 5-minute intervals, and the degree of induced muscle contraction was measured in each case. Using a Dual Impedance Research Stimulator (Harvard Apparatus, USA) and bipolar electrode, stimulations of 20 Hz and 70 Hz (3 V, 0.3 ms) were applied to the tibial nerves for 5 seconds in order to induce gastrocnemius muscle contraction under physiological (20 Hz) and pathological (70 Hz) conditions. Incomplete tetanus was induced by stimulation of 20 Hz, and complete tetanus was induced by 70 Hz. A 20 Hz stimulation was applied first, and 70 Hz stimulation was then applied after a 5-minute rest, to prevent muscle fatigue [9]. Electrical stimulations were applied before medicine injection, 1 hour after the injection, and 2 hours after the injection.

Silk threads were attached to the Achilles tendon and connected to a force transducer (Harvard Apparatus, USA), and the degree of muscle contraction induced in the gastrocnemius muscle by 20 Hz or 70 Hz electrical stimulation of the tibial nerve was measured. Then, based on the degree of muscle contraction before and at 1 and 2 h after the injection of water-extracted Jakyak-Gamcho decoction or 70% ethanol-extracted Jakyak-Gamcho decoction, muscle cramp reduction rates were compared (1). The thread was connected to the gastrocnemius muscle uniformly in each rat, with respect to its anatomical position. The force transducer was connected to MP100A-CE system (BIOPAC System Inc., USA), and the degree of muscle contraction was visualized and analyzed via Acqknowledge 3.9.1 software. Consider the following:

(1) Reduction  rate=1−tension  1 h  or  2 h  after  stimulationtension  before  stimulation×100%.

### 2.5. Statistical Analysis

Based on tension measurements before and after medicine injection, muscle cramp reduction rates were calculated and used in analyses. IBM SPSS Statistics 21 (IBM, USA) was used for statistical analyses. A normality test and equal variation assumption were satisfied, and reduction rates among groups were compared via ANOVA. Duncan's test was used for multiple comparisons. The significance level was set at *p* < 0.05.

## 3. Results

### 3.1. HPLC

**Figure 2:**
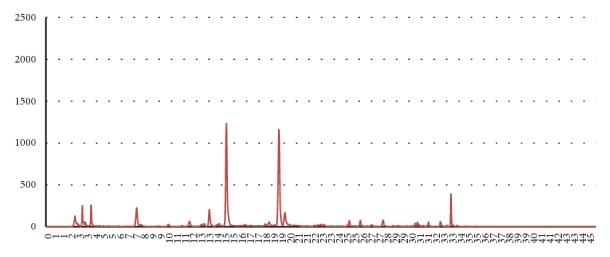
Chromatogram derived from a water-extracted Radix Glycyrrhizae/Radix Paeoniae Jakyak-Gamcho decoction.

**Figure 3:**
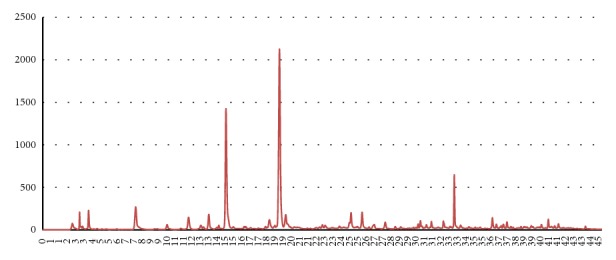
Chromatogram derived from a 70% ethanol-extracted Radix Glycyrrhizae/Radix Paeoniae Jakyak-Gamcho decoction.

HPLC results are shown in Figures 2 and 3 and Table 2. Larger amounts of all of the effective index components including paeoniflorin, benzoic acid, glycyrrhizin, and isoliquiritin were extracted using 70% ethanol as an extractant, as compared to water. Compared to the amounts derived via water, 70% ethanol yielded 68.216 *μ*g/mL more paeoniflorin, 0.091 *μ*g/mL more benzoic acid, 208.540 *μ*g/mL more glycyrrhizin, and 56.705 *μ*g/mL more isoliquiritin. Notably, the extraction yields of glycyrrhizin and isoliquiritin, effective index components of Radix Glycyrrhizae, were 1.6 and 2.6 times greater, respectively, when using 70% ethanol as the extractant instead of water.

### 3.2. Initiating Frequency

**Figure 4:**
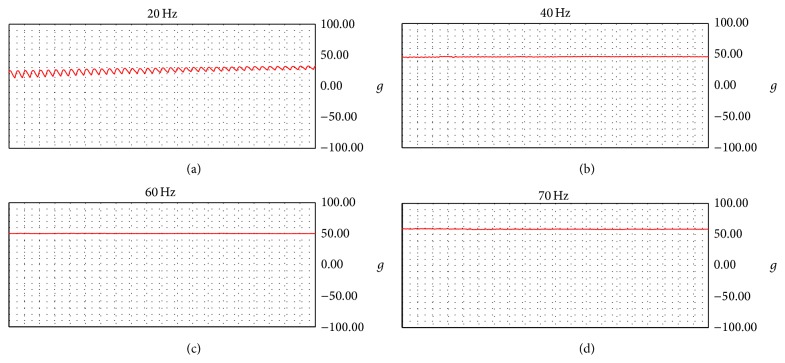
Initiating frequency.

Figure 4 shows the results of the preliminary study. Electrical stimulation of 20 Hz yielded a serrated contraction curve, and the contraction curves gradually became flatter as the intensity of stimulation was increased. In addition, the degree of muscle contraction increased with stronger electrical stimulation, and complete tetanus was induced at 70 Hz.

### 3.3. Muscle Cramp Reduction Rate

**Figure 5:**
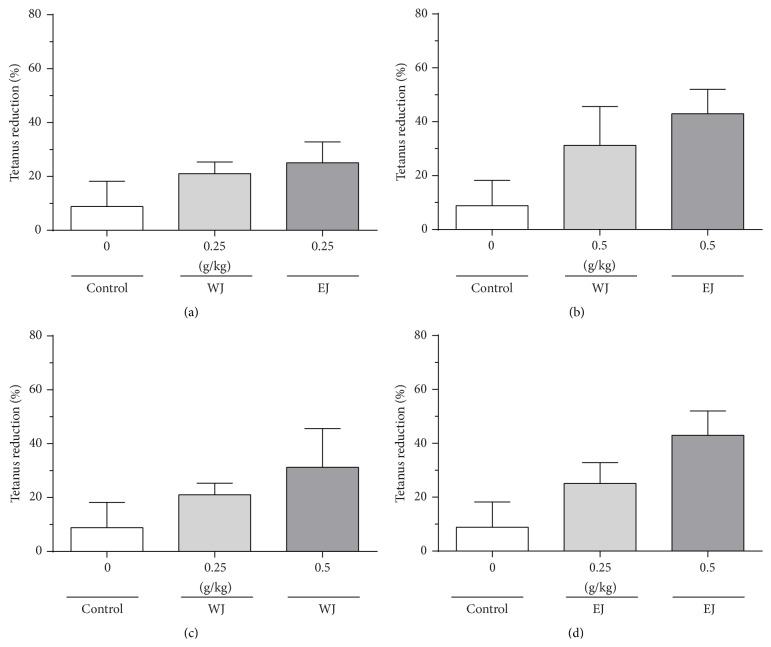
Muscle cramp reduction rates with electrical stimulation of 20 Hz 2 hours after medicine injection, by solvent (a, b) and medicine dose (c, d). Each bar represents mean + SEM of 5 animals.

Compared to the time before medicine injection, muscle cramp reduction rates 1 hour after injection did not differ significantly at electrical stimulations of 20 Hz or 70 Hz in any group (data not shown). The degrees of muscle contraction at 20 Hz before medicine injection and 2 hours after medicine injection were compared, and the reduction rates are shown in Figure 5. The solvent used did not have a significant effect on muscle cramp reduction rate (Figures 5(a) and 5(b)) and neither did the medicine dose (Figures 5(c) and 5(d)).

**Figure 6:**
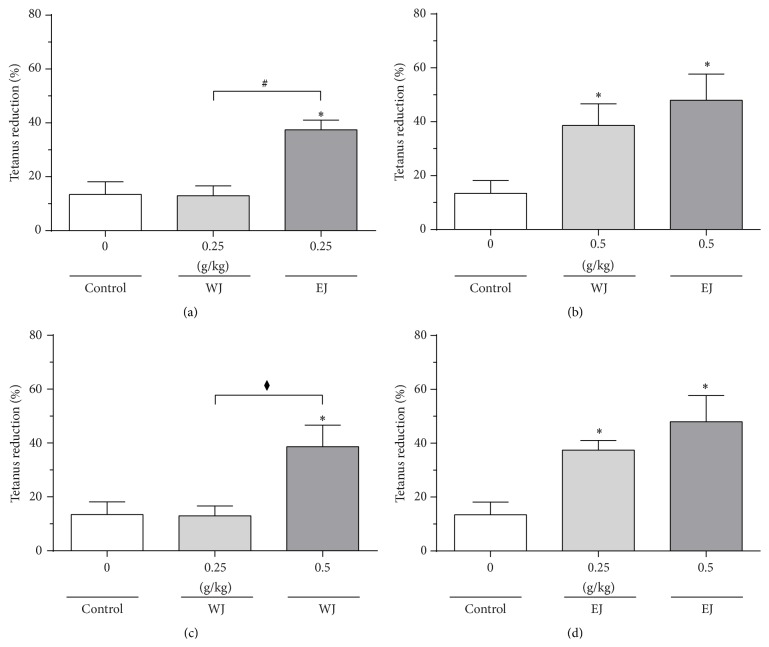
Muscle cramp reduction rates with electrical stimulation of 70 Hz 2 hours after medicine injection, by solvent (a, b) and medicine dose (c, d). Each bar represents mean + SEM of 5 animals. ^*∗*^ *p* < 0.05 compared to the control group by ANOVA and Duncan's test. ^#^ *p* < 0.05; comparison between the WJ and EJ groups by Duncan's test. ^⧫^ *p* < 0.05; comparison between the 0.25 g/kg and 0.5 g/kg WJ groups by Duncan's test.

The degrees of muscle cramping at 70 Hz before medicine injection and 2 hours after medicine injection were compared and the reduction rates are shown in Figure 6. With regard to water-extracted Jakyak-Gamcho decoction, 0.25 g/kg did not yield a significant spasmolytic effect compared to the control group (distilled water) (*p* > 0.05). Conversely, 0.25 g/kg of 70% ethanol-extracted Jakyak-Gamcho decoction yielded a significant spasmolytic effect compared to both the control group and 0.25 g/kg of water-extracted Jakyak-Gamcho decoction (*p* < 0.05 for both comparisons) (a). At the higher dose of 0.5 g/kg, both water-extracted and 70% ethanol-extracted Jakyak-Gamcho decoctions yielded significant reductions in muscle cramping, as compared to the control group (*p* < 0.05 for both comparisons) (b).

With regard to medicine dose, 0.25 g/kg of water-extracted Jakyak-Gamcho decoction did not yield a significant reduction in the rate of muscle cramping compared to the control group (distilled water) (*p* > 0.05). On the other hand, 0.5 g/kg exhibited a significant spasmolytic effect compared to both the 0.25 g/kg group and the control group (*p* < 0.05 for both comparisons) (c). Both 0.25 g/kg and 0.5 g/kg of 70% ethanol-extracted Jakyak-Gamcho decoctions yielded significant spasmolytic effects compared to the control group (*p* < 0.05 for both comparisons) (d).

## 4. Discussion

Muscle cramping is a condition that occurs suddenly, and the duration of it is short [6]. In this regard, muscle cramps are hard to predict and hard to manage. Therefore, preventive approaches that can reduce the degree and frequency of unpredicted muscle cramps are required rather than administration of therapeutic agents every time they occur. Thus, in order to investigate muscle cramp prevention, Jakyak-Gamcho decoctions were injected 1 hour prior to the experimental induction of cramping in this study. We designed an intuitive muscle cramp induction model to use in this study. As each individual rat possesses different muscle force, it is not appropriate to simply compare the muscle cramp-induced gastrocnemius muscle tension between different groups of rats. Therefore, in this study gastrocnemius muscle tension was measured before and after injection; then these measurements were used to calculate reduction rates. By controlling for the individual differences in muscle capacity in each rat, muscle cramp reduction rates were able to be compared via groups, and significant outcomes were obtained.

While the spasmolytic mechanisms of Jakyak-Gamcho decoctions are not clearly understood, it has been suggested that paeoniflorin desensitizes nicotine acetylcholine receptors, and by acting together with glycyrrhizin, contractile Ca^2+^ mobilization is inhibited and neuromuscular transmission is blocked [11]. In this current study, the amounts of all four effective index components were higher in 70% ethanol-extracted Jakyak-Gamcho decoction than water-extracted Jakyak-Gamcho decoction, as determined by HPLC analysis. This is consistent with the results of a previous study investigating the extraction efficiency of Jakyak-Gamcho decoctions [17]. Particularly, among the constituents of Jakyak-Gamcho decoction, Radix Glycyrrhizae has significant spasmolytic activity [9]. In this current study, the yields of the Radix Glycyrrhizae constituents glycyrrhizin and isoliquiritin were 1.6 and 2.6 times higher, respectively, when extracted with 70% ethanol than when extracted with water. This confirmed that different extractants can yield different amounts of active components related to the spasmolytic effects of Jakyak-Gamcho decoctions.

In herbal medicines, intracellular contents from which active components are derived are extracted through cell walls or by breaking down cell walls [19, 20]. In the case of ethanol extraction in herbal medicine, ethanol solvents break down the cell walls of medicinal ingredients, facilitating the extraction of active components from within the cells [21, 22]. In the present study, higher amounts of the active components of Jakyak-Gamcho decoction were extracted by 70% ethanol than by water. We suggest that this is because ethanol breaks down the cell walls of the ingredients of oriental medicines, ultimately enhancing extraction efficiency.

Since ethanol can explode when heated, a traditional boiling method was not used in the extraction of Jakyak-Gamcho decoction with ethanol in this study. Instead, ultrasound-reflux extraction, which is known to be effective for herbal medicines [19, 20], was used. To ensure an accurate comparison between the two solvents investigated, the same ultrasound-reflux extraction method was used for the water-extracted Jakyak-Gamcho decoction.

Several studies have investigated the threshold frequency at which complete tetanus starts to develop and continue [23–25]. However, the threshold frequency at which complete tetanus reportedly started in these previous studies differed from the initiating frequency observed in this current study [9]. This is likely due to differences in experimental conditions and inconsistent operational definitions of complete tetanus. As muscle cramping is an instant musculoskeletal condition and is accompanied by instant intense pain, we designed an electrical stimulation experiment that can induce maximum muscle contraction instantly in spite of its short duration. Accordingly, an experiment investigating initiating frequency was performed, and complete tetanus was confirmed to develop at 70 Hz.

Neither the muscle contraction rates induced by electrical stimulation with 20 Hz nor those induced by 70 Hz were reduced significantly by any of the decoctions in any group, 1 hour after the injection of medicine. This might have been because glycyrrhetinic acid, which is glycyrrhizin's hydrolysis product, had not yet been absorbed sufficiently to yield an effect. Glycyrrhetinic acid is a major component for spasmolytic effect [9]. It has been reported that the highest plasma concentration of glycyrrhetinic acid is observed 8–10 hours after oral administration [26]. Although intraduodenal administration can increase the rate of absorption, 1 hour might not be enough to yield a spasmolytic effect. Two hours after injection, muscle cramps induced by electrical stimulation of 20 Hz were not significantly reduced, indicating that Jakyak-Gamcho decoctions were not effective in the muscle contraction of incomplete tetanus, which occurs before complete tetanus. Conversely, a significant difference was observed when an electrical stimulation of 70 Hz was applied, suggesting that Jakyak-Gamcho decoctions can be effective for muscle cramp alleviation associated with complete tetanus. However, further studies are needed to elucidate the pharmacological mechanism of Jakyak-Gamcho decoction, which makes it effective as a spasmolytic agent in case of pathological muscle cramps but not for physiological muscle contraction.

With regard to electrical stimulation of 70 Hz that was applied 2 hours after medicine injection, 0.25 g/kg of water-extracted Jakyak-Gamcho decoction did not show a significant spasmolytic effect compared to distilled water, but 0.25 g/kg of 70% ethanol-extracted Jakyak-Gamcho decoction did. Even at an equivalent dose (0.25 g/kg), the effects of 70% ethanol-extracted Jakyak-Gamcho decoction were more significant than those of water-extracted Jakyak-Gamcho decoction (*p* < 0.05) and therefore the difference in the amounts of effective index components evidently results in different effects in practice. Only when 0.5 g/kg of water-extracted Jakyak-Gamcho decoction was injected did it exhibit a spasmolytic effect similar to that of 0.25 g/kg of 70% ethanol-extracted Jakyak-Gamcho decoction. With regard to 70% ethanol-extracted Jakyak-Gamcho decoctions, differences between the effects of 0.25 g/kg and 0.5 g/kg were not significant (*p* > 0.05), which may be because 0.25 g/kg achieves the maximum possible level of effectiveness of Jakyak-Gamcho decoction [27, 28].

Limitations of this study include that an electrical stimulation differs from general muscle cramp causes. Electrical stimulation-induced muscle cramps may possess different physiological characteristics to general muscle cramps. In addition, the surgery was invasive, which may have led to additional differences between the physiological conditions in the study and those of naturally occurring muscle cramps. However, direct muscle tension measurement is required in order to measure muscle cramps, so invasive surgery procedures were unavoidable.

In the present study, we increased the efficiency of Jakyak-Gamcho extraction by using an alternative extractant to water and confirmed that this corresponded with an increase in the spasmolytic effectiveness of Jakyak-Gamcho decoction in an animal model. To date, Jakyak-Gamcho decoctions have been shown to be effective for muscle relaxation and muscle pain alleviation [9, 12–14, 29]. In this current* in vivo* study, ethanol-extracted Jakyak-Gamcho decoction was superior to water-extracted Jakyak-Gamcho decoction with regard to both extraction efficiency and spasmolytic effects. The elucidation of changes in the dosages of herbal medicines in the form of powders is required, for the quantitative standardization of their active components. The results of this current study suggest changes in extraction methods when Jakyak-Gamcho decoctions are prepared and converted into powder form. Moreover, the results suggest that it may be worthwhile to investigate alternative extraction methods in terms of extraction efficiency and* in vivo* effectiveness for other herbal medicines besides Jakyak-Gamcho decoction.

## 5. Conclusion

This study confirmed that differences in the amounts of effective index components in Jakyak-Gamcho decoctions resulted in differences in their effects* in vivo*. Jakyak-Gamcho decoction extracted with 70% ethanol exhibited higher amounts of effective index components than that extracted with water. In addition, 2 hours after medicine injection, the injection of 70% ethanol-extracted Jakyak-Gamcho decoction, which contained relatively higher amounts of effective index components, was associated with greater reductions in muscle cramping. The results of this study suggest that it may be worthwhile to investigate alternative extraction methods in terms of extraction efficiency and* in vivo* effectiveness for other herbal medicines in the future.

## Figures and Tables

**Table 1:** Gradient details.

Time (min)	A (%)	B (%)
0	90	10
5	90	10
25	70	30
35	40	60
45	5	95
48	5	95
49	90	10
60	90	10

**Table 2:** HPLC quantification results.

	Water-extracted decoction	70% ethanol-extracted decoction
Acquired amount (*µ*g/mL)		
Paeoniflorin	383.626	451.842
Benzoic acid	17.664	17.755
Isoliquiritin	33.729	90.434
Glycyrrhizin	343.904	555.444
